# Subpopulations of hypocretin/orexin neurons differ in measures of their cell proliferation, dynorphin co-expression, projections, and response to embryonic ethanol exposure

**DOI:** 10.1038/s41598-023-35432-w

**Published:** 2023-05-25

**Authors:** Nushrat Yasmin, Adam D. Collier, Olga Karatayev, Abdul R. Abdulai, Boyi Yu, Milisia Fam, Nailya Khalizova, Sarah F. Leibowitz

**Affiliations:** Laboratory of Behavioral Neurobiology, The Rockefeller University, 1230 York Avenue, New York, NY 10065 USA

**Keywords:** Neural circuits, Addiction, Cellular neuroscience, Axon and dendritic guidance, Neuronal development

## Abstract

Numerous studies in animals demonstrate that embryonic exposure to ethanol (EtOH) at low-moderate doses stimulates neurogenesis and increases the number of hypothalamic neurons expressing the peptide, hypocretin/orexin (Hcrt). A recent study in zebrafish showed that this effect on the Hcrt neurons in the anterior hypothalamus (AH) is area specific, evident in the anterior (aAH) but not posterior (pAH) part of this region. To understand specific factors that may determine the differential sensitivity to EtOH of these Hcrt subpopulations, we performed additional measures in zebrafish of their cell proliferation, co-expression of the opioid dynorphin (Dyn), and neuronal projections. In association with the increase in Hcrt neurons in the aAH but not pAH, EtOH significantly increased only in the aAH the proliferation of Hcrt neurons and their number lacking Dyn co-expression. The projections of these subpopulations differed markedly in their directionality, with those from the pAH primarily descending to the locus coeruleus and those from the aAH ascending to the subpallium, and they were both stimulated by EtOH, which induced specifically the most anterior subpallium-projecting Hcrt neurons to become ectopically expressed beyond the aAH. These differences between the Hcrt subpopulations suggest they are functionally distinct in their regulation of behavior.

## Introduction

Exposure to ethanol (EtOH) early in development is found in both animal and clinical studies to produce long-lasting neuronal changes that are accompanied by multiple behavioral disturbances, including an increase in alcohol consumption along with behaviors such as arousal, anxiety and motivation studies^1,2^. Embryonic EtOH exposure is known to produce differential effects that vary depending on the concentration of EtOH and brain area, with high doses often decreasing neurogenesis and increasing apoptosis in certain brain regions^3,4^ and low-to-moderate doses stimulating neurogenesis with no effect on apoptosis in areas such as the hippocampus and hypothalamus^2,5–7^. Also, EtOH at low-moderate concentrations increases in both rats and zebrafish the number of hypothalamic neurons expressing various peptides including hypocretin/orexin (Hcrt)^8,9^, which itself is known to promote alcohol intake and the related behaviors^10^. In a recent study^11^, we discovered in zebrafish that EtOH induced an increase in Hcrt neurons in the anterior hypothalamus (AH) and is anatomically specific within this area, occurring only in the anterior (aAH) but not posterior (pAH) part of this region. We also found that EtOH causes Hcrt neurons to be ectopically expressed anterior to the hypothalamus, within the preoptic area (POA) of zebrafish and also in anterior brain regions of rats^9^, consistent with evidence that neurons cluster in various ectopic brain locations of children with fetal alcohol spectrum disorders (FASD)^12,13^. With no studies showing brain-region specific effects on the proliferation of Hcrt neurons, we investigated here whether the EtOH-induced increase in number of Hcrt neurons in the aAH and their ectopic expression in the POA is due to an increase in their proliferation in these areas. With Hcrt implicated in many aspects of alcohol drinking and addiction^14,15^, its role in these behaviors is found to be affected by various peptides and neurotransmitters that colocalize within Hcrt neurons, including the opioid peptide, dynorphin (Dyn), leading us to also examine here the effects of EtOH on this peptide. With Dyn found to be co-expressed in most of the Hcrt neurons in both zebrafish^16^ and rodents^17^, this raises the question as to whether EtOH exposure affects the expression of Dyn within the Hcrt neurons, consequently altering their behavioral function. Although there are no studies testing the effects of embryonic EtOH exposure on Dyn in zebrafish, its effects on Dyn expression in rats is found to vary across brain areas, with Dyn expression decreased in the nucleus accumbens core^18^ while increased in the ventral tegmental area^19^. Like Hcrt, Dyn is shown to have a role in alcohol drinking behaviors^20^, elevated in the hypothalamus of rats selectively bred for alcohol preference and high anxiety^21^. However, unlike Hcrt which is excitatory and associated with reward and arousal^22^, Dyn is inhibitory and implicated in depressive-like states^23^, suggesting that any change in its expression level within Hcrt neurons is likely to alter their behavioral functioning.

In addition to the anatomically specific effects of embryonic EtOH on Hcrt neurons, we recently found that embryonic EtOH exposure increases the number of processes emanating from the soma of Hcrt neurons in the AH of zebrafish and similarly in the lateral hypothalamus of rats^9^. Other studies in rats demonstrate that EtOH’s effect on neuronal morphology and projections is brain-area specific, with EtOH increasing dendrite length and complexity in the hippocampus and dorsal medial striatum^24–26^ while decreasing these measures in the accumbens shell and prefrontal cortex^25,27^, consequently producing structural changes in the neuronal circuits^28^. These effects which would alter Hcrt signaling and neurocircuitry likely contribute to the subsequent behavioral changes induced by developmental EtOH exposure in animals^29,30^, as indicated by the behavioral deficits in children with FASD^31^.

Building on this evidence, we further investigated here these specific measures of the aAH and pAH Hcrt subpopulations and ectopic POA neurons and the effects of embryonic EtOH exposure on their neuronal development that may contribute to the associated disturbances in behavior. Specifically, we tested whether the pAH, aAH and POA Hcrt neurons differ under control conditions and after EtOH exposure in their: 1) cell proliferation activity; 2) pattern of Dyn expression within these Hcrt neurons; and 3) projections throughout the brain that innervate functionally relevant brain areas.

## Materials and methods

### Animals and housing

Transgenic *Hcrt:EGFP*^29^ zebrafish (*Danio rerio)* of the AB strain background were used in this study. Adult zebrafish were group-housed in 3 L tanks (Aquatic Habitat, Apopka, FL) with recirculating water flow at a temperature between 28–29 °C and a pH between 6.9–7.4 as previously described^32^. All breeding and raising of embryos occurred within an AAALAC accredited facility using protocols approved by the Rockefeller University Institutional Animal Care and Use Committee and guidance of the NIH Guide for the Care and Use of Laboratory Animals. The study was carried out in compliance with the ARRIVE guidelines.

## Embryonic ethanol treatment

Embryonic exposure of zebrafish to EtOH was performed, as described in our previous reports^10,27^ and briefly summarized here. At 22 h post-fertilization (hpf), embryos were removed from an incubator and placed in a solution of either 0.0% (control) or 0.5% (vol/vol %) EtOH for 2 h, washed in fresh embryo medium, and then returned to the incubator. This concentration of EtOH represents a low-to-moderate concentration and does not produce physical dysmorphologies often reported at higher concentrations, thus providing a model of the highly prevalent alcohol-related neurodevelopmental disorder^33,34^.

### EdU labeling

To evaluate the immediate effects of embryonic EtOH on proliferation, actively proliferating cells were labelled using Click iT EdU Cell Proliferation Kit (Invitrogen, C10337) as previously described^30,35^. Immediately after EtOH exposure, zebrafish at 24 hpf were incubated for 30 min in a 10 mM EdU solution (15% DMSO in embryo water), first on ice for 5 min and then at room temperature for 25 min. They were then returned to a 28.5 °C incubator and raised to 6 dpf when the Hcrt neurons become clearly expressed in distinct subpopulations within the aAH, pAH and POA. The EdU-labeled cells observed at 6 dpf indicate the cells that were actively proliferating at 24 hpf when the EdU was administered. The fish were then fixed overnight in 4% PFA and rinsed with PBS. The brains were then removed and incubated at room temperature for 3 h in EdU reaction mixture, followed by a 2 h incubation in blocking solution (2% normal donkey serum in 0.1% PBST). They were then incubated overnight at 4 °C in rabbit anti-GFP primary antibody (Life Technologies Corp, A-11122, 1:200), followed by 3 × 15 min wash in 0.1% PBST, and then incubated for 3 h in a solution of 4,6-Diamidino-2-phenylindole (DAPI, 1:5000) and Alexa 488 anti-rabbit secondary antibody (Abcam, ab150073, 1:400).

### RNAscope in situ hybridization and immunofluorescence

We used the RNAscope Multiplex Fluorescent Reagent Kit v2 (ACD Bio-Techne, catalog # 323,100) to detect pDyn transcripts in 6 dpf *Hcrt:EGFP* zebrafish brains using the pDyn probe (ACD Bio-Techne, catalog # 865,971-C3). This procedure was performed using a combination of the manufacturer’s protocol and protocols described in recent publications^36,37^. Briefly, isolated brains from previously dehydrated fish were rehydrated in a 75–50–25% methanol-0.1% PBST series and fixed for at least 5 h in 4% PFA (in 1% Tween-20) at 4 °C. The samples were rinsed 3 times with 0.1% PBST and treated with Protease III for 15 min in a 40 °C water bath and washed again with 0.1% PBST followed by an overnight hybridization with the probe at 40 °C. The brains were then washed and post-fixed with 4% PFA for 10 min at room temperature. All subsequent incubations were done at 40 °C, with 3 × 15 min washes using 0.2 × SSCT between each step. They were then incubated with AMP1 (30 min), AMP2 (15 min) and AMP3 (30 min) in this order. To develop the signal, the brains were incubated with RNAscope Multiplex FL v2 HRP-C3 for 15 min, stained with Opal 690 (Akoya Biosciences FP1497001KT) in 1:1500 dilution in TSA buffer for 30 min, and blocked in the RNAscope Multiplex FL v2 HRP blocker for 15 min. Finally, they were incubated for 30 min at 4 °C in DAPI (1:200) diluted in 0.2% PBST, washed 3 × 5 min with PBS, and stored at 4 °C in fresh PBS until imaging. Due to the extensive tissue processing required by this procedure, we were unable to perform triple-labeling using EdU, RNAscope, and immunofluorescent labeling.

### Microscopy and image analysis

The EdU and RNAscope brain samples of *Hcrt:EGFP* zebrafish at 6 dpf were imaged with a 40 × objective lens on an inverted Zeiss LSM 780 laser scanning confocal microscope, beginning from the dorsal side of the brain, and then analyzed in Imaris 9.9.1 software. The AH was divided into equal halves along the AP axes into regions defined as posterior AH (pAH) and anterior AH (aAH). The number of Hcrt:EGFP neurons in the pAH and aAH and further anterior within the POA was quantified by manually counting using the “Spots” function in Imaris. The accurate counting of individual Hcrt neurons was confirmed by ensuring that each neuron corresponded with nuclear DAPI staining. The density of EdU labeling was quantified by determining, using Imaris “Spot” function with 6 µm spot size, the number of EdU labeled cells within the pAH, aAH and POA and dividing these values by the volume of each respective region. The number of Hcrt:EGFP neurons with EdU labeling was determined through manual quantification. In the RNAscope images, the density of pDyn transcripts was quantified using the “Spots” function with a 1 µm spot size, determined by measuring the diameter of these transcripts and then normalized to the volume exported from the “surface” of each brain region in Imaris. To measure pDyn transcripts colocalizing with Hcrt:EGFP neurons, we first applied Imaris “surface” to mask these neurons and used the “Spot colocalize with surface” function in Imaris (spot distance threshold of 1 µm) to ensure these transcripts were in fact colocalized within the masked Hcrt:EGFP neurons. The Hcrt:EGFP neurons were defined as “Hcrt with no pDyn” if they had 0–3 pDyn transcripts and as “Hcrt with pDyn” if they had at least 4 transcripts per neuron.

To acquire images of Hcrt neuronal projections throughout the brain, transgenic 6 dpf *Hcrt:EGFP* zebrafish were live-imaged using confocal microscopy, with a 25 × objective lens on a Zeiss LSM 780 laser scanning confocal microscope and a z step of 1.0 μm. We first created 3D reconstructions of neuronal projections from Hcrt neurons located in the pAH, aAH and POA using the “Filaments” function of Imaris^38^. Spot size for start point and seeding points of the filament was 7 µm and 0.9 µm with a threshold of 1350 and 350, respectively, followed by manual correction. We then quantified the average neuronal projection length (sum of the lengths of all projection segments for each Hcrt neuron), the number of branch points (sum of branching points of all projection segments for each Hcrt neurons), number of terminal points (sum of the number of end points of each projection from each Hcrt neuron), and number of sholl intersections (number of projection intersections from each Hcrt neuron through concentric spheres with a 1 µm step resolution) from the total projections of Hcrt neurons within the pAH, aAH and POA.

**Figure 1:**
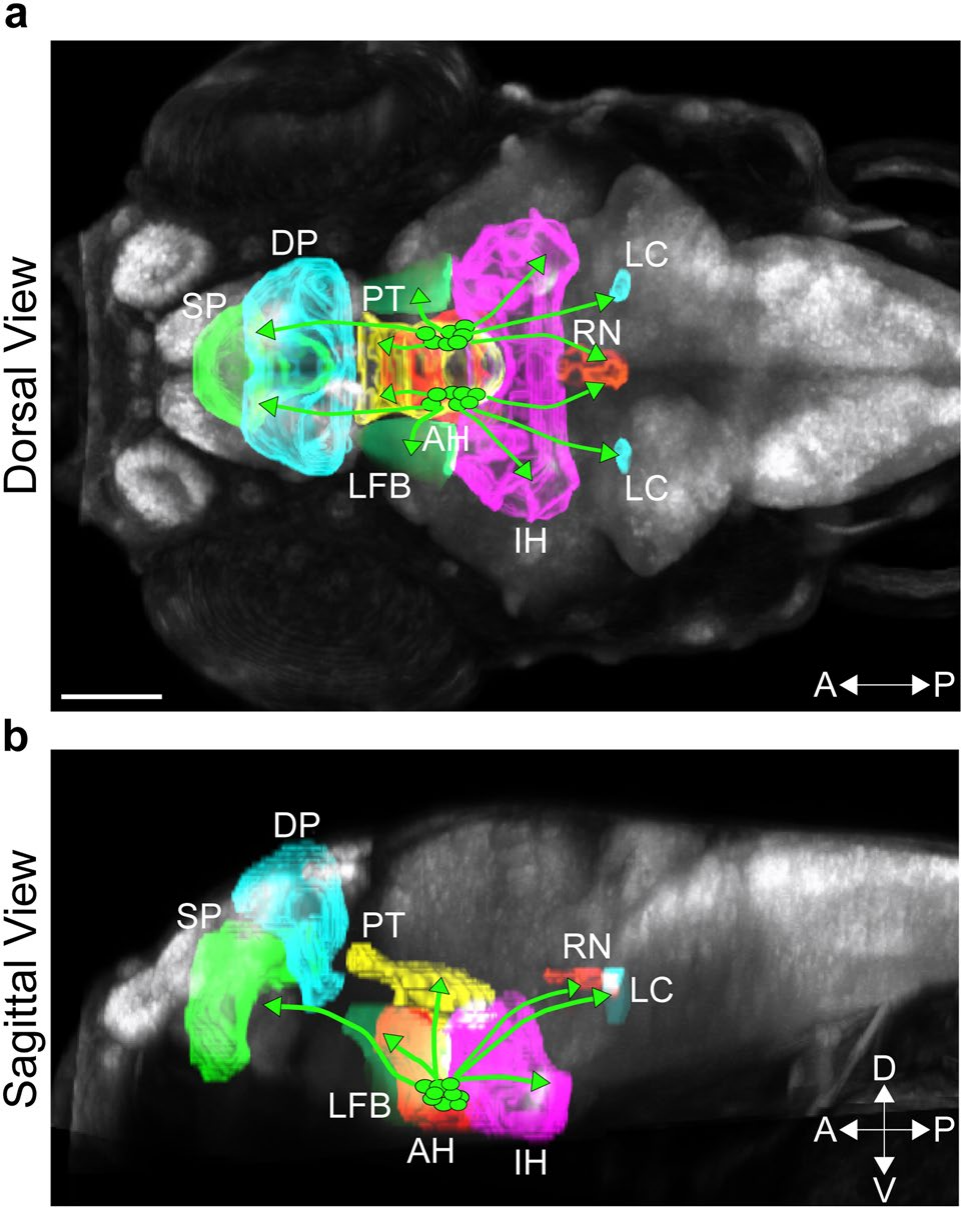
Photomicrographs illustrating brain regions of interest innervated by Hcrt neurons in a 6 dpf zebrafish brain. Brain regions derived from the Zebrafish Brain Browser^33^ are illustrated by different colors within a representative zebrafish brain, shown from a dorsal view (**a**) and sagittal view (**b**). Brain regions of interest from anterior to posterior include the SP (green), DP (light blue) and PT (yellow) in the anterior brain, AH (red), IH (pink) and LFB (light green) within the hypothalamic area, and the LC (light blue) and RN (orange) in the posterior brain. Hcrt neurons are represented by circles (green) within the AH, with lines from these neurons representing their projections to the different brain regions. Scale bar: 100 µm. Abbreviations: aAH: anterior part of the anterior hypothalamus, pAH: posterior part of the anterior hypothalamus, POA: preoptic area, SP: subpallium, DP: dorsal pallium, PT: posterior tuberculum, IH: intermediate hypothalamus, LFB: lateral forebrain bundle, RN: raphe nucleus, LC: locus coeruleus, Hcrt: hypocretin, dpf: days post fertilization, A: anterior, P: posterior, D: Dorsal, V: ventral.

To quantify terminal points and branch points within brain areas of interest, we first used the “Surface” function of Imaris to mask out functionally relevant brain regions that are known to be innervated by Hcrt neurons, as described^39,40^. In addition to the pAH, aAH and POA, these included the lateral forebrain bundle (LFB), posterior tuberculum (PT), intermediate hypothalamus (IH), locus coeruleus (LC), raphe nucleus (RN), subpallium (SP), dorsal pallium (DP), as illustrated in Fig. 1. For this analysis, the Hcrt projections to the furthest anterior regions including olfactory bulb, pineal gland and optic tectum and those to the most posterior regions beyond the LC including the spinal cord were excluded. The anatomy for each brain region was derived from the Zebrafish Brain Browser atlas, following previously published guidelines^41^. The density of branch points and terminal points were calculated within each of these brain areas by quantifying number of branch points and terminal points and then dividing these values by the volume (exported from Imaris “Surface” function) of each respective region. Sholl analysis was unable to be performed within brain regions, as the beginning point of each projection, namely the cell body, is needed to be present within the region of analysis.

### Statistical analyses

All Hcrt, EdU, and pDyn and were analyzed using two-way ANOVA, which tested the main effects of EtOH and brain area and their interaction, followed by post-hoc Hold-Sidak’s multiple comparisons test. Data derived from total projections and those within specific brain regions was analyzed by unpaired *t* tests with the Holm-Sidak multiple comparisons correction as necessary. Since there were no Hcrt neurons evident in the POA of control fish, the POA data were omitted from each two-way ANOVA where the neurons were analyzed. All tests were two-tailed, and significance was determined at *p* < 0.05. All data were analyzed using Prism (version 9, GraphPad, San Diego, CA) and are presented as mean ± SEM in the figures and tables.

## Results

### Embryonic EtOH exposure stimulates the proliferation of Hcrt neurons in the aAH and ectopic POA but not pAH

**Figure 2:**
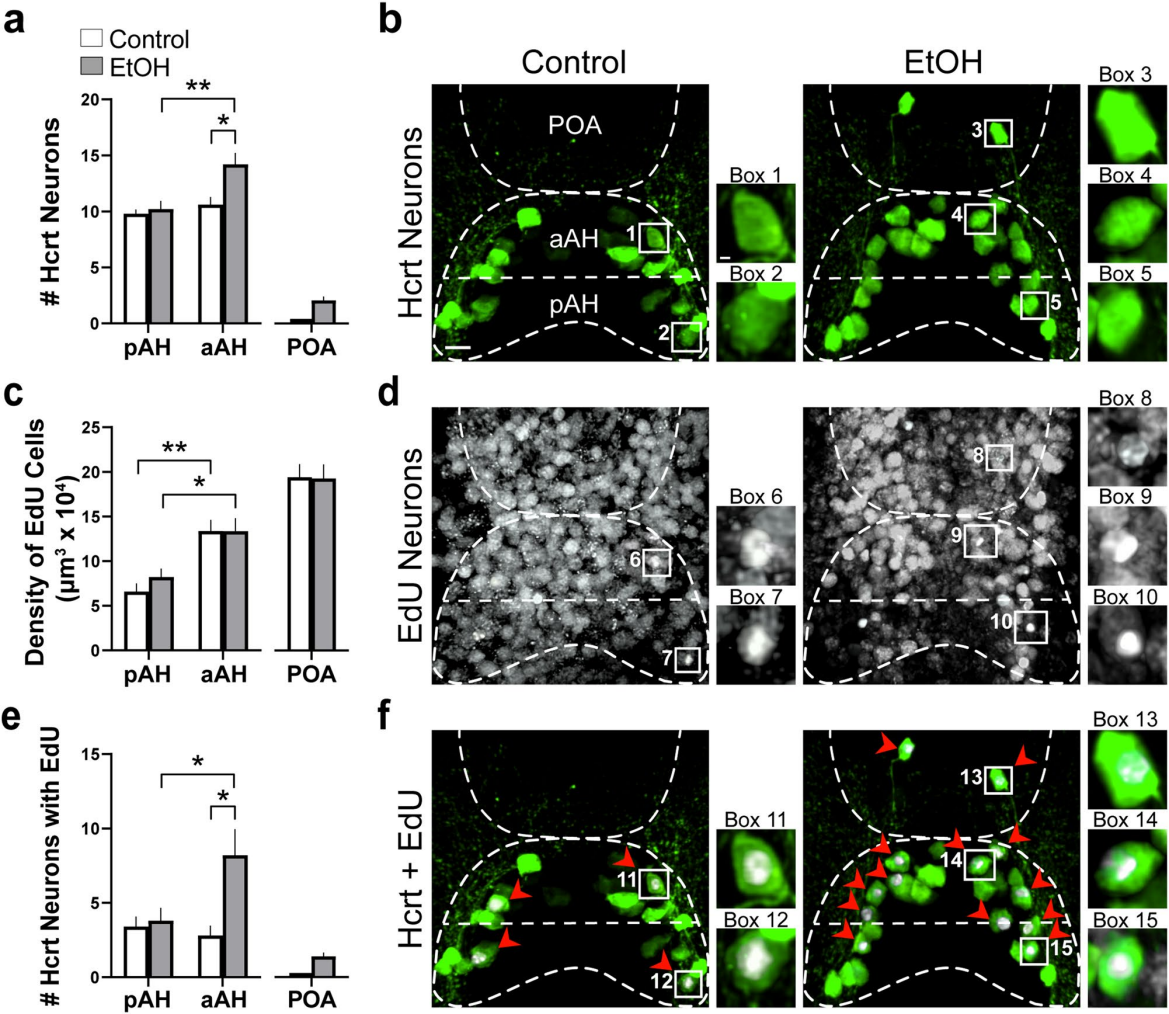
Effects of exposure to embryonic EtOH (0.5% v/v, 22–24 hpf) on the number of Hcrt neurons, the density of EdU cells, and the number of Hcrt neurons co-expressing EdU within the pAH, aAH and POA of 6 dpf transgenic *Hcrt:EGFP* zebrafish brains. (**a**) Bar graphs (n = 5/group) show that EtOH increases the number of Hcrt neurons in the aAH but not the pAH relative to control, causes the number in the aAH to be greater than in the pAH of EtOH-treated zebrafish, and induces a few ectopic Hcrt neurons (2.2 ± 0.374) further anterior in the POA that are not detected in control zebrafish. (Two-way ANOVA, EtOH main effect: (*F* (1, 16) = 7.339, *p* = 0.016), Brain area main effect: (*F* (1, 16) = 10.57, *p* = 0.005), EtOH x Brain area interaction: (*F* (1, 16) = 4.697, *p* = 0.046), followed by Holm-Sidak post-hoc described in the text). (**b**) Photomicrographs (40×, dorsal view) illustrate Hcrt neurons (green) after immunofluorescence staining that are located in the pAH and aAH of control and EtOH-treated zebrafish and are ectopically expressed in the POA of EtOH-treated zebrafish. Boxes 1–2 show enlargements of single Hcrt neurons in the aAH and pAH of control, respectively, and boxes 3–5 show enlargements of Hcrt neurons in the POA, aAH, and pAH of EtOH, respectively. (**c**) Bar graphs (n = 5/group) show that the density of EdU cells in the aAH is greater than the density in the pAH of both control and EtOH zebrafish, and EtOH has no effect on the density of EdU-expressing cells in the pAH, aAH or POA. (Two-way ANOVA, EtOH main effect: EtOH main effect (*F* (1, 24) = 0.199, *p* = 0.660), Brain area main effect: (*F* (2, 24) = 43.03, *p* < 0.0001), EtOH x Brain area interaction: (*F* (2, 24) = 0.291, *p* = 0.751), Holm-Sidak post-hoc test described in text). (**d**) Photomicrographs (25×, dorsal view) show EdU labeling (white) in the pAH, aAH and POA of control and EtOH zebrafish. Boxes 6–7 show enlargements of EdU cells located in the aAH and pAH, respectively, of control zebrafish, and boxes 8–10 show enlargements of EdU cells located in the POA, aAH, and pAH of EtOH-treated zebrafish, respectively. (**e**) Bar graphs (n = 5/group) show that EtOH increases the co-expression of Hcrt with EdU in the aAH but not the pAH, and in the EtOH-treated zebrafish there are more Hcrt neurons co-expressing EdU in the aAH than the pAH. Ectopic POA Hcrt neurons are also found to co-express EdU in EtOH-treated zebrafish (1.4 ± 0.245). (Two-way ANOVA, EtOH main effect: (*F* (1, 16) = 7.188, *p* = 0.164), Brain area main effect: (*F* (1, 16) = 3.085, *p* = 0.0981), EtOH x Brain area interaction: (*F* (1, 16) = 5.342, *p* = 0.420), followed by Holm-Sidak post-hoc described in the text). (**f**) Photomicrographs (25×, dorsal view) illustrate an overlay of Hcrt (green) and EdU labeling (white) and their co-expression. Boxes 11–12 show enlargements of single Hcrt neurons co-expressing EdU in the pAH and aAH of control, respectively, and boxes 13–15 show enlargements of single Hcrt neurons co-expressing EdU in the POA, aAH, and pAH, respectively. Scale bars: low magnification 10 µm; high magnification 2 µm. All results are shown as means ± standard errors. Abbreviations: EtOH: ethanol, aAH: anterior part of the anterior hypothalamus, pAH: posterior part of the anterior hypothalamus, POA: preoptic area, Hcrt: hypocretin, EdU: 5–ethynyl–2′–deoxyuridine, hpf: hours post fertilization, dpf: days post fertilization.

We first tested in 6 dpf zebrafish, using the proliferation marker EdU, if the anatomically specific, stimulatory effect of embryonic EtOH exposure on Hcrt neurons in the aAH subpopulation is attributed to an increase in the proliferation of these Hcrt neurons. Analysis of *Hcrt:EGFP* zebrafish showed that the number of Hcrt neurons, similar in the aAH and pAH under control conditions (*p* = 0.838), was significantly increased by EtOH in the aAH (*p* = 0.0132) but not the pAH (*p* = 0.914) (Fig. 2a), as illustrated in the photomicrographs (Fig. 2b). This increase in number of aAH neurons, which was evident throughout the entire aAH, caused this area to have significantly more Hcrt neurons than the pAH (*p* = 0.007), and it was accompanied by the appearance after EtOH of a few (2–3) ectopic Hcrt neurons immediately anterior to the aAH in the POA (Fig. 2a, b), consistent with our recent study^9^. A difference between the aAH and pAH areas was also revealed by analysis of the density of EdU-labeled cells. While unaffected by EtOH in the aAH (*p* = 0.984) and pAH (*p* = 0.567), their density was significantly greater in the aAH than the pAH both under control conditions (*p* = 0.013) and after EtOH treatment (*p* = 0.047) (Fig. 2c, d), indicating that cells in the aAH are undergoing more active proliferation. The density of single-labeled EdU cells within the POA, which was also unaffected by EtOH compared to control (*p* = 0.9952), was even higher than in the aAH of control (*p* = 0.026) and EtOH (*p* = 0.023) fish.

A further difference between the aAH and pAH subpopulations was revealed by analysis of the Hcrt neurons that co-expressed EdU. Compared to control, EtOH significantly increased their number in the aAH (*p* = 0.017) but not the pAH (*p* = 0.910), and these double-labeled neurons, evenly distributed throughout the aAH, were greater in number than in the pAH (*p* = 0.043) (Fig. 2e–f). The percent of Hcrt neurons co-expressing EdU in the aAH was increased from 26 to 56% (t (8) = 2.45, *p* = 0.039) while unaffected in the pAH (t (8) = 0.187, *p* = 0.857), and the percent of EdU cells co-expressing Hcrt was also increased from 4 to 16% in the aAH (t (8) = 2.43, *p* = 0.041) while unchanged in the pAH (t (8) = 0.516, *p* = 0.620), underscoring the anatomical specificity of EtOH’s effect. In the POA, a majority of the EtOH-induced ectopic Hcrt neurons (averaging 2.2 ± 0.37) was also found to co-express EdU (1.4 ± 0.245) (Fig. 2e–f), indicating they are actively proliferating. Together, these findings that EtOH increases the proliferation specifically of Hcrt neurons in the aAH and ectopic POA neurons just anterior to the aAH, without having any effect on the overall density of single-labeled EdU cells in these areas, suggests that the Hcrt neurons are particularly sensitive to EtOH’s stimulatory effect on their proliferation.

### Embryonic EtOH exposure increases the number of Hcrt neurons without pDyn in the aAH but not pAH

We next tested the effect of embryonic EtOH exposure on the peptide, pDyn, and its pattern of co-expression in the subpopulation of aAH and pAH Hcrt neurons. As in the first experiment, analysis of the Hcrt neurons showed that EtOH increased their number throughout the entire aAH (*p* = 0.0001) but not in the pAH (*p* = 0.631), causing them to be significantly greater in the aAH than pAH of EtOH (*p* < 0.0001) but not control (*p* = 0.827) zebrafish (Supplementary Table, S1). Analysis of pDyn in these areas, in contrast, revealed no effect of EtOH compared to control on the density of pDyn transcripts in both the aAH (*p* = 0.576) and pAH (*p* = 0.618) and also in the POA (*p* = 0.256) where a few ectopic Hcrt neurons (averaging 2.2 ± 0.31) were once again detected (Supplementary Table, S1).

**Figure 3:**
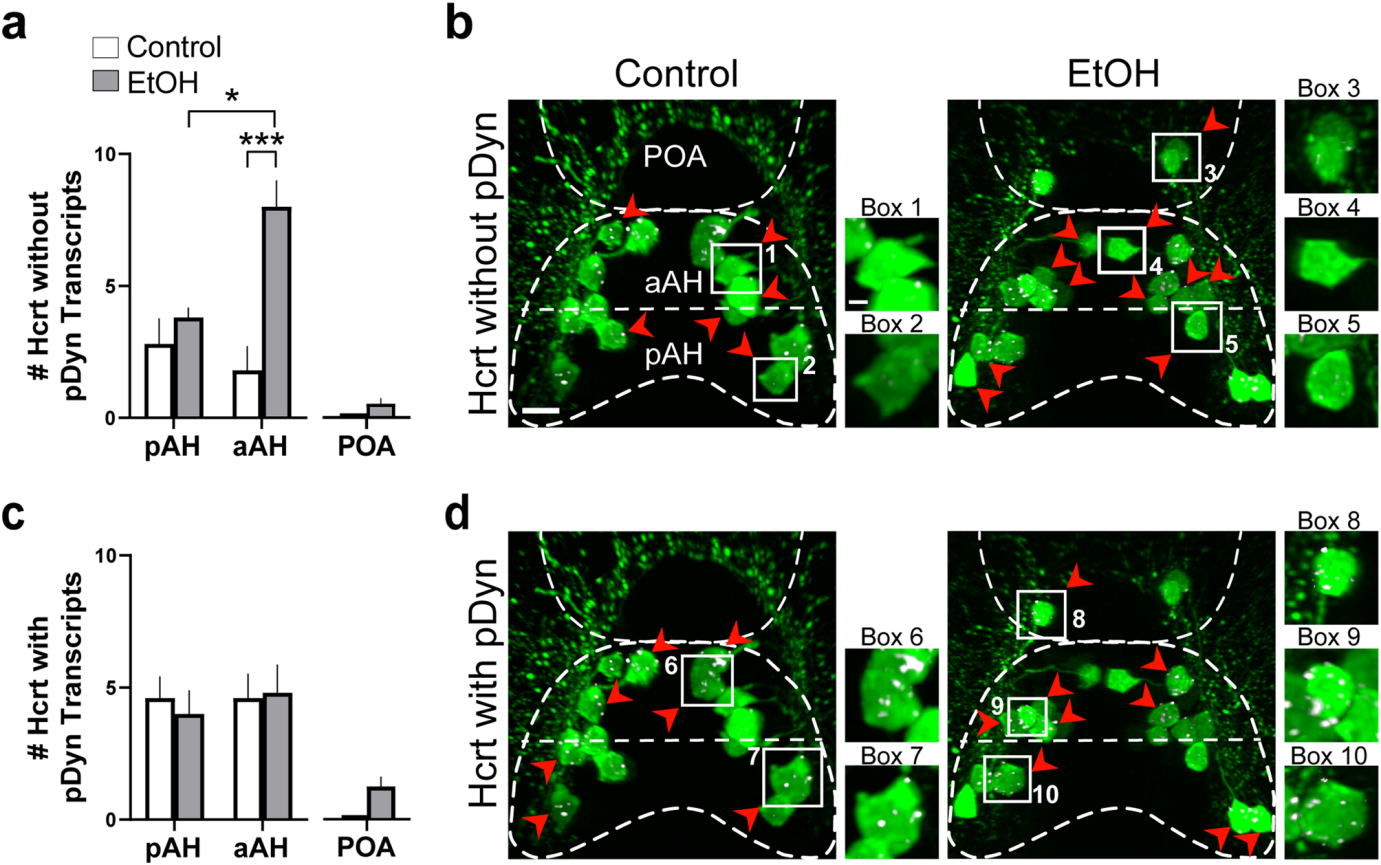
Effects of exposure to embryonic EtOH (0.5% v/v, 22–24 hpf) on the number of Hcrt neurons with and without co-expression of transcripts of the neuropeptide pDyn within the pAH, aAH and POA of 6 dpf transgenic *Hcrt:EGFP* zebrafish brains. (**a**) Bar graphs (n = 5/group) show that EtOH increases the number of Hcrt neurons with no pDyn expression in the aAH but not the pAH, causing the aAH to have a greater number of Hcrt neurons with no pDyn expression than the pAH and inducing an ectopic Hcrt neuron (0.6 ± 0.245) in the POA of EtOH-treated zebrafish having no pDyn expression. (Two-way ANOVA, EtOH main effect: (*F* (1, 16) = 17.75, *p* = 0.0007), Brain area main effect: (*F* (1, 16) = 3.507, *p* = 0.0794), EtOH x Brain area interaction: (*F* (1, 16) = 9.260, *p* = 0.0078), followed by Holm-Sidak post-hoc described in the text). (**b**) Photomicrographs (40×, dorsal view) illustrate Hcrt neurons (green) after immunofluorescence and RNAscope staining with no pDyn (white) expression, as indicated by red arrows. Boxes 1–2 show enlargements of single Hcrt neurons with no pDyn expression located in the aAH and pAH of control, respectively, and boxes 3–5 show enlargements of Hcrt neurons with no pDyn expression located in the POA, aAH, and pAH of EtOH-treated zebrafish, respectively. (**c**) Bar graphs (n = 5/group) show that EtOH compared to control has no effect on the number of Hcrt neurons with pDyn expression in the pAH or aAH and induces some (1.2 ± 0.374) ectopic Hcrt neurons in the POA of EtOH treated zebrafish having pDyn expression. (Two-way ANOVA, EtOH main effect: treatment (*F* (1, 16) = 0.046, *p* = 0.833), Brain area main effect: (*F* (1, 16) = 0.1850, *p* = 0.6729), EtOH x Brain area interaction: (*F* (1, 16) = 0.1850, *p* = 0.6729), followed by Holm-Sidak post-hoc described in the text). (**d**) Photomicrographs (40×, dorsal view) illustrate Hcrt neurons (green) after immunofluorescence and RNAscope staining with pDyn (white) expression, as indicated by red arrows. Boxes 1–2 show enlargements of single Hcrt neurons with pDyn expression located in the aAH and pAH of control, respectively, and boxes 3–5 show enlargements of Hcrt neurons with pDyn expression located in the POA, aAH, and pAH of EtOH-treated zebrafish, respectively. Scale bars: low magnification 10 µm; high magnification 2 µm. All results are shown as means ± standard errors. Abbreviations: EtOH: ethanol, pDyn: prodynorpin, aAH: anterior part of the anterior hypothalamus, pAH: posterior part of the anterior hypothalamus, POA: preoptic area, Hcrt: hypocretin, hpf: hours post fertilization, dpf: days post fertilization.

A further analysis of the co-expression of pDyn transcripts in the Hcrt neurons revealed that the aAH and pAH areas were similar under control conditions with most of their Hcrt neurons co-expressing pDyn but differed markedly after EtOH exposure, as illustrated in the photomicrographs (Fig. 3a–d). In the aAH, EtOH compared to control, while having no effect on the number of Hcrt neurons co-expressing pDyn transcripts (*p* = 0.999), unexpectedly significantly increased the number of Hcrt neurons that did *not* co-express pDyn (*p* = 0.0003). These Hcrt neurons without pDyn transcripts were evenly distributed throughout the entire aAH, and their percent of the total population in this area was markedly increased from 28 to 63% (*p* = 0.047). This is in contrast to the pAH, where EtOH had no effect on the number of Hcrt neurons that did (*p* = 0.999) or did not (*p* = 0.999) co-express pDyn transcripts and on their percent with (*p* = 0.148) or without (*p* = 0.134) pDyn co-expression. In the POA, the levels of co-expression in the few ectopic Hcrt neurons (averaging 2.2 ± 0.31) were mixed with 30% lacking pDyn (0.6 ± 0.245) and the others co-expressing pDyn (1.2 ± 0.374) (Fig. 3a–d). Thus, while having no effect on the overall density of pDyn transcripts in these areas and the number of Hcrt neurons with pDyn transcripts, EtOH had a specific, stimulatory effect only in the aAH on the number of Hcrt neurons lacking pDyn co-expression.

### Embryonic EtOH exposure stimulates long projections from Hcrt neurons in the pAH and Hcrt neurons that are ectopically expressed in the POA

**Figure 4:**
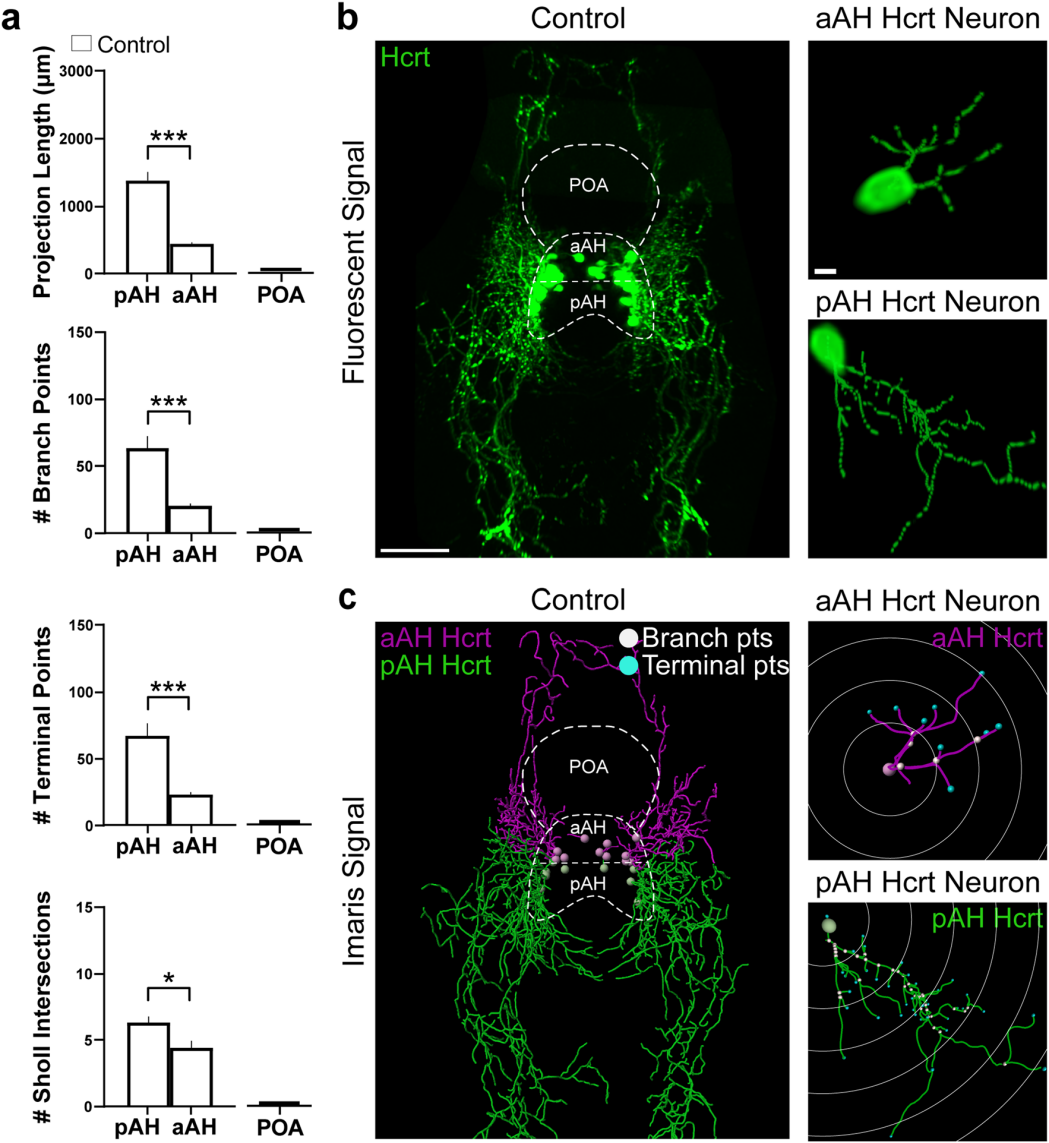
Characterization under control conditions of the projections from the Hcrt neuron subpopulations in the pAH and aAH of 6 dpf transgenic *Hcrt:EGFP* zebrafish brains, along with an illustration of the Imaris software methodology used to quantify the branching, terminal points and sholl intersections of these projections. (**a**) Bar graphs (n = 5/group) show in control fish that the projections from pAH Hcrt neurons compared to those from aAH neurons have greater projection length and number of branch points, terminal points, and sholl intersections, with no Hcrt neurons or projections detected in the POA as indicated by flat bars in the graphs. (**b**) Photomicrograph (25×, dorsal view) obtained using confocal microscopy illustrates in a control *Hcrt:EGFP* zebrafish brain a representative image of the Hcrt neurons and projections (green) from the subpopulations, with the pAH, aAH and POA areas outlined by dashed lines and enlargements of a single representative Hcrt neuron along with its projections observed in the aAH (top) and pAH (bottom). (**c**) A digital representation created from the same confocal image using the “Filaments” function of Imaris software shows that the projections from Hcrt neurons located in the pAH (green) are primarily descending in the posterior direction, while the projections from Hcrt neurons located within the aAH (magenta) are primarily ascending in the anterior direction. The measures of projection length and number of branch points (white), terminal points (blue), and sholl intersections are derived from an analysis using Imaris “spots” of this digital reconstruction. Enlargements show with Imaris software the projections of a single representative Hcrt neuron located in the aAH (top) and pAH (bottom), with the white concentric circles starting at the projection point of origin of the Hcrt soma extending outwards to demonstrate the sholl intersection analysis, with the projection intersections through these circles used to measure the spatial distribution of these projections. Scale bars: low magnification 50 µm; high magnification 10 µm. Abbreviations: aAH: anterior part of the anterior hypothalamus, pAH: posterior part of the anterior hypothalamus, POA: preoptic area, Hcrt: hypocretin, dpf: days post fertilization.

This experiment examined the projections originating from the subpopulations of aAH and pAH Hcrt neurons and the ectopically expressed Hcrt neurons in the POA, to determine if their projections differ under control conditions as well as in response to EtOH exposure. Our analysis of all projections from the aAH and pAH neurons under control conditions (Fig. 4a–c) showed that, while many of the projections from these two subpopulations were short in length (< 90 µM from their soma point of origin) and terminated in areas within or adjacent to the hypothalamus, others were long in length (> 90 µM from their soma point of origin) and terminated in specific areas distant from the hypothalamus. Whereas the short projections overlapped extensively, the long projections from the subpopulations differed markedly in their directionality, with those from aAH Hcrt neurons projecting in the anterior direction and those from pAH neurons projecting in the posterior direction. They also differed in their morphology, with the projections from pAH Hcrt neurons compared to aAH neurons being longer (*p* < 0.0001) and having more branch points (*p* = 0.0019), terminal points (*p* = 0.0017), and sholl intersections (*p* = 0.0237).

**Figure 5:**
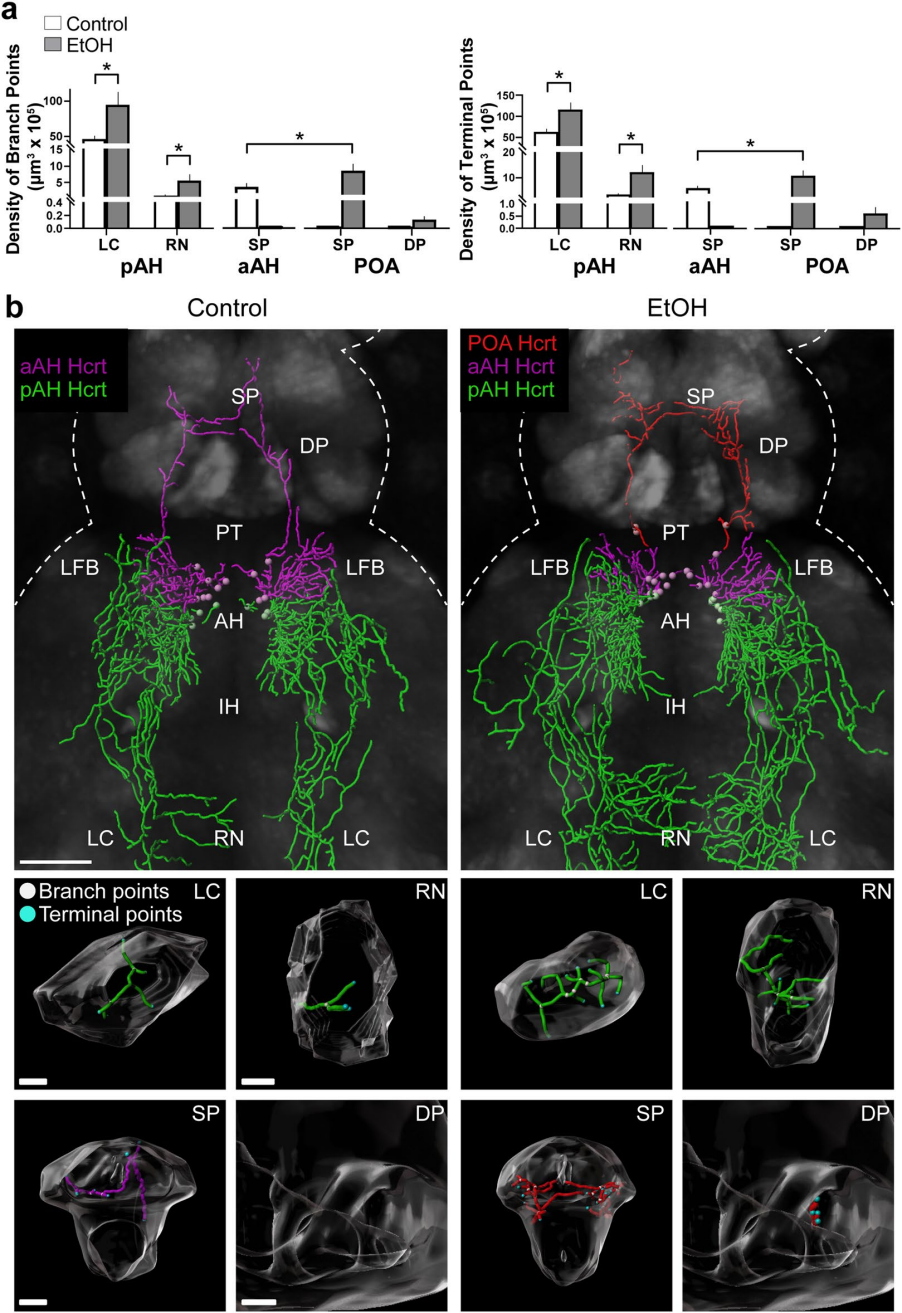
Effects of embryonic exposure to EtOH (0.5% v/v, 22–24 hpf) on the density in specific brain areas of branch points and terminal points of the long projections from Hcrt neurons in the pAH, aAH and POA of 6 dpf transgenic *Hcrt:EGFP* zebrafish brains. (**a**) Bar graphs (n = 5/group) show that EtOH increases the density of branch points (left) and terminal points (right) of the long descending projections from the pAH Hcrt neurons to the LC and RN. EtOH also stimulates the long ascending projections to the SP from aAH Hcrt neurons that are evident in control but not after EtOH exposure, causing them to become ectopically expressed in the POA and increasing the density of their branch points (left) and terminal points (right) in the SP while also inducing branch points and terminal points in the DP. The data for analysis of the short Hcrt projections, to areas within the hypothalamus (pAH, aAH and IH) and adjacent to this structure (LFB and PT), are described in the text and presented in Supplementary Table, S2. The absence of aAH projection data in the SP of the EtOH condition and POA projection data in the SP and DP of the control condition are indicated by flat bars in the graphs (**b**) Photomicrographs (25×, dorsal view) illustrate Imaris software representations of projections from Hcrt neurons in the pAH (green) and aAH (magenta) of both control and EtOH-treated zebrafish and from Hcrt neurons ectopically located in the POA (red) of EtOH-treated zebrafish, with branch points indicated by white dots and terminal points indicated by blue dots. Enlargements of the LC, RN, SP and DP found to be innervated by the long projections are presented below, illustrating in control and EtOH fish the branch points (white) and terminal points (blue) of the projections from Hcrt neurons in the pAH (green) and aAH (magenta) in control and EtOH fist and from the POA (red) in EtOH fish. Scale bars: low magnification 50 µm; LC: 10 µm; RN: 15 µm; SP: 20 µm; DP: 20 µm. All results are shown as means ± standard errors. Abbreviations: EtOH: ethanol, aAH: anterior part of the anterior hypothalamus, pAH: posterior part of the anterior hypothalamus, POA: preoptic area, SP: subpallium, DP: dorsal pallium, PT: posterior tuberculum, IH: intermediate hypothalamus, LFB: lateral forebrain bundle, RN: raphe nucleus, LC: locus coeruleus, Hcrt: hypocretin, hpf: hours post fertilization, dpf: days post fertilization.

Our analysis of the Hcrt projections as they relate to the specific brain areas they innervate showed that EtOH had little or no effect on their short projections within or close to the hypothalamus (Supplementary Table, S2). This includes the projections from aAH neurons that had their terminal points mostly in the aAH as well as the LFB and PT and also the projections from pAH neurons that had their terminal points mostly in the pAH and IH and also in the LFB and PT where a small increase in density of branch points (*p* = 0.049 and *p* = 0.009, respectively) and terminal points (*p* = 0.040 and *p* = 0.011, respectively) was observed. In contrast to their short projections, analysis of the long projections from aAH and pAH Hcrt neurons to distant brain areas revealed marked differences in control fish along with similar stimulatory effects in EtOH fish. Under control conditions (Fig. 5a, b), the long-projecting pAH neurons were evenly distributed throughout the pAH and had descending projections with almost all of their terminal points in the LC and a few in the RN, while the long-projecting aAH neurons were clustered in the most anterior region of the aAH close to the border of the POA and had ascending projections with all of their terminal points in the SP and none in the DP. Embryonic EtOH exposure stimulated the long projections from pAH neurons, increasing both their projection branch points in the LC (*p* = 0.023) and RN (*p* = 0.025) and their projection terminal points in the LC (*p* = 0.0178) and RN (*p* = 0.0124) (Fig. 5a, b). It also stimulated the long projections from the most anterior aAH neurons, which became ectopically expressed immediately anterior within the POA and had ascending projections terminating in the SP where EtOH increased the density of their branch points (*p* = 0.036) and terminal points (*p* = 0.025) compared to these measures of the long aAH projections in control (Fig. 5a, b). These projections from the EtOH-induced ectopic neurons appeared to extend further dorsally, with their branch points (0.163/um^3^ × 10^4^ ± 0.067) and terminal points (0.599/um^3^ × 10^4^ ± 0.523) also detected in the DP. Thus, while having little effect on the short projections from the pAH and aAH Hcrt neurons, EtOH stimulated both the long descending projections to the LC and RN from pAH neurons and the long ascending projections to the SP and DP from the most anterior aAH neurons that became ectopically expressed.

## Discussion

In addition to confirming our recent finding in zebrafish that embryonic EtOH at low-moderate doses increases the number of Hcrt neurons in the aAH but not pAH and induces ectopic Hcrt neurons anterior to the aAH within the POA^11^, this report provides new evidence suggesting that this anatomically-specific increase in Hcrt number is due to an increase in cell proliferation, indicated by a greater number of EdU-labeled Hcrt neurons. This is consistent with other findings showing that EtOH in rats stimulates neurogenesis in an anatomically specific manner, increasing the proliferation of enkephalin-expressing neurons specifically in the core but not shell of the nucleus accumbens^8,2^. Also, EtOH in zebrafish induces ectopic expression of oxytocin^42^ and cranial neural crest cells^43^ and also causes the formation of hindbrain heterotopies of facial branchial motor neurons^4^. Our finding here that EtOH stimulates the proliferation of Hcrt neurons, while having no effect on the total number of EdU-labeled cells as shown earlier^30^, suggests that the Hcrt neurons are particularly sensitive to EtOH’s stimulatory effect on proliferation. This effect which occurs specifically in the aAH may be related to our additional finding that the aAH undergoes more active proliferation than the pAH, both under control conditions and after EtOH exposure. The importance of this naturally higher level of cell proliferation for revealing the EtOH-induced increase in Hcrt neurons in the aAH is further suggested by our finding that the POA where ectopic EdU-expressing Hcrt neurons are evident also exhibits more active cell proliferation. This may also explain similar findings in rats, showing that prenatal EtOH exposure induces ectopic Hcrt neurons in areas anterior to the hypothalamus, including the nucleus accumbens core and ventromedial caudate putamen^9^. These EtOH-induced increases in the number of Hcrt neurons are supported by the Hcrt reserve hypothesis, which proposes that the Hcrt system naturally exhibits dynamic shifts in peptide expression through a pool of reserve Hcrt neurons that permits adaptive behaviors and also allows these neurons to be recruited and upregulated in pathological states such as after drug exposure^44^. Published studies in rodents show that most Hcrt neurons co-express Dyn^17^ and prenatal EtOH exposure has mixed or no effects on Dyn expression in different brain areas^19,45,46^. While a majority of the Hcrt neurons under control conditions are found here to co-express pDyn in larval zebrafish as described in adults^47^, our results show no effect in either the aAH or pAH of embryonic EtOH exposure on the total number of pDyn transcripts or the number of Hcrt neurons co-expressing pDyn. The main, unexpected finding here is that EtOH increases the number and percent of Hcrt neurons that do *not* co-express pDyn and this effect again occurs exclusively in the aAH. Also important is our finding that these Hcrt neurons lacking pDyn are similar to those co-expressing EdU after EtOH, both in their number and anatomical distribution throughout the aAH, suggesting they are the same subpopulation. The anatomical specificity of this EtOH effect is further supported by our result showing that the Hcrt neurons in the pAH are unaffected by EtOH regardless of whether they co-express pDyn, similar to the lack of effect on the proliferation of pAH Hcrt neurons. While the mechanism underlying the EtOH-induced increase in Hcrt neurons that do not co-express pDyn is unknown, it is possible that the lack of pDyn in aAH Hcrt neurons is creating a more permissive environment for an EtOH-induced increase in proliferation, with studies showing that Dyn administration has a suppressive effect both in vivo and in vitro on neurogenesis and the differentiation of rodent neural stem cells^48,49^. It is also worth noting that Hcrt neurons contain other peptides in addition to pDyn^39,50^ that may also be affected by EtOH, possibly through a compensatory mechanism, as has been previously described for Dyn expression after Hcrt knockdown^51^. Although Dyn and Hcrt are co-transmitted from the same vesicles^52^, these peptides are found to have an inverse relationship in rodents, with Hcrt being excitatory and associated with reward and arousal and a stimulation of dopamine neurons^52,53^ and Dyn being inhibitory and implicated in depressive-like states and an inhibition of dopamine^54,55^. Thus, these EtOH-induced aAH Hcrt neurons, which lack pDyn and are increased in number, are likely functionally altered with an increase in excitatory signaling.

Prior studies in zebrafish of projections from the entire population of Hcrt neurons have described a dense cluster of projections that innervate regions throughout the brain and are mostly descending from the hypothalamus to the mesencephalon^56,57^, similar to that described in rodents^58^. While we recently reported that embryonic EtOH exposure increases the number of processes emanating from the soma of normally located Hcrt neurons in rats as well as zebrafish^9^, the present report is the first to characterize the projections from specific aAH and pAH subpopulations of Hcrt neurons and demonstrate how EtOH affects these projections. Prior studies in rodents have shown that EtOH’s effects on neuronal projections vary depending on the anatomical location of the neurons, with prenatal EtOH exposure increasing the number, length, and dendritic complexity of projections from neurons in the hippocampus^24,25^ while decreasing these measures of projections from neurons in the prefrontal cortex^59,60^. They can also vary within a brain region, with EtOH decreasing dendritic length and branching of neurons in the shell but not core of the nucleus accumbens^27^ and differentially affecting the projections from pyramidal neurons across different layers of the medial prefrontal cortex^61^. Here we demonstrate that projections from the subpopulations of aAH and pAH Hcrt neurons differ markedly under control conditions. The projections from pAH neurons descending primarily to the LC but also the RN are longer and have greater branching, terminal points and sholl intersections compared to those from aAH neurons which ascend almost exclusively to the SP. This is in line with evidence showing that projections from posterior and anterior subpopulations of hypothalamic oxytocin neurons in zebrafish innervate very different regions, with the former projecting to multiple brain areas and the latter projecting to the pituitary^62^. Our finding that EtOH has little or no effect on the short projections from aAH and pAH neurons while markedly stimulating the long projections from both subpopulations, agrees with evidence that developmental EtOH exposure differentially alters the short and long projections from pyramidal neurons in layer VI of the medial prefrontal cortex^61^. While increasing the number of Hcrt neurons only in the aAH, EtOH stimulates the long projections from both subpopulations to very different brain regions, increasing the branching and terminal points of the descending projections from pAH neurons that terminate in the LC and RN and also the branching and terminal points of the ascending projections from aAH neurons that become ectopically expressed further anterior in the POA and terminate in the SP. This effect on projections of ectopic neurons agrees with studies showing that EtOH induces fibers of ectopic cranial nerves^63^ and stimulates ectopic intracortical connectivity that actually persists into an F3 generation^64^. In addition to increasing projection branching and terminal points, EtOH appears to alter these long projections from the ectopic Hcrt neurons so that they also innervate the DP, a region dorsal to the SP reported in adult zebrafish to be homologous to the mammalian amygdala and hippocampus^65^. While there is evidence for Hcrt receptors in the telencephalon of larval and adult zebrafish^66,67^ and the innervation of the DP by Hcrt neurons in adult fish^68^, studies of larval zebrafish have yet to reveal Hcrt neuronal projections to the DP^39^, suggesting that EtOH may be causing premature maturation of this Hcrt neurocircuitry similar to that reported for hippocampal pyramidal neurons^25^.

While the mechanisms underlying EtOH’s anatomically specific effects on the development of Hcrt neuronal circuitry remain to be characterized, there is strong evidence suggesting that the Cxcl12/Cxcr4 chemokine system plays an important role. This chemokine system is stimulated by embryonic EtOH exposure^69^ and shown to be involved in mediating the stimulatory effect of EtOH on the proliferation of neurons^70,71^, with pretreatment of the Cxcr4 antagonist AMD3100 found to block the EtOH-induced increase in aAH Hcrt neurons and formation of ectopic Hcrt neurons in the POA^11^. There is also evidence that the Cxcl12/Cxcr4 system mediates the anatomically specific stimulation of Hcrt neurons in the aAH, acting through a natural posterior-to-anterior concentration gradient that is stimulated by EtOH and has levels lowest in the pAH where Hcrt neurons are unaffected by EtOH, higher in the aAH where they are increased by EtOH, and highest further anterior where EtOH induces the ectopic neurons. Another factor possibly involved in mediating EtOH’s anatomically specific, stimulatory effect on Hcrt neurons is the peptide Dyn, which is found to decrease the expression of the Cxcr4 receptor^72,73^. With the lack of pDyn specifically in aAH Hcrt neurons after EtOH, their levels of Cxcr4 expression are likely to increase and be involved in promoting their migration further anterior into the POA where Cxcl12 levels also become higher along the gradient. With additional studies showing that Cxcl12 modulates dendritic spine morphology, increases spine density, and stimulates axonal branching^74–77^, this chemokine may also have a role in EtOH’s stimulatory effects on the Hcrt neuronal projections. There is evidence that it guides projections through the brain to their terminal sites, with disruption of Cxcl12 expression found to produce abnormally located GnRH3 neuron projections^78^, ectopic habenular axonal projections^79^, and disturbed axonal pathfinding of olfactory sensory neurons^80^. Thus, Cxcl12 may contribute to the EtOH-induced stimulation of projections from the ectopic Hcrt neurons not only to the SP but also into the DP.

In zebrafish^30,39^ as well as rodents^10^, Hcrt neurons are known to have a positive relationship to alcohol intake and related behaviors such as locomotion and anxiety, and these behaviors in turn are shown to be stimulated by embryonic EtOH exposure along with the increase in Hcrt neurons. The functional importance of specific subpopulations of Hcrt neurons in mediating these behaviors is suggested by results from our recent report in zebrafish^9^, showing that ablation of the ectopic POA Hcrt neurons or the Hcrt neurons in the most anterior part of the aAH blocks the EtOH-induced increase in anxiety-like behavior, and ablation of the ectopic Hcrt neurons but not the aAH Hcrt neurons blocks the EtOH-induced increase in locomotor behavior. While these ablation findings along with evidence showing 0.5% EtOH to increase the number of differentiated neurons in zebrafish hypothalamus^81^ suggest that these neurons are mature and functional, further validation of this could be provided by studies showing the stage of the neuronal cell maturation cycle that each of these Hcrt neurons have reached when examined at 6 dpf. Further evidence for the differential, functional role of specific subpopulations is provided in rats, showing that the motivation to seek cocaine is reduced by knockdown of Hcrt neurons in the lateral hypothalamus but not the medial perifornical area^82^. Here we demonstrate that EtOH increases specifically in the aAH the proliferation of Hcrt neurons and their number lacking pDyn co-expression, suggesting that these Hcrt neurons with elevated levels of excitatory Hcrt relative to inhibitory Dyn signaling have distinct behavioral functions. This includes the promotion of reward-related behaviors in rodents^52^ that may be related to the increase in alcohol sipping observed in clinical adolescent populations exposed prenatally to EtOH^83^. This is supported by a recent study in mice^84^, showing that optogenetic activation of neurons expressing both Hcrt and Dyn produces reward seeking behavior and accumbens dopamine release and these effects are blocked by administration of an Hcrt receptor antagonist but not a Dyn receptor antagonist.

While the direct impact on behavior of the EtOH-induced changes in Hcrt neuronal projections is unknown, there is strong evidence from studies of the FASD syndrome that abnormal circuit formation in the cortex decreases behavioral flexibility and hyperactivity^85^ and changes in the morphology of neuronal projections in the prefrontal cortex and hippocampus cause marked deficits in cognition^61,86^. Here we show that the EtOH-induced ectopic Hcrt neurons from the aAH almost exclusively innervate the SP, a brain area that is known to contain Hcrt receptors and to be homologous to the mammalian basal ganglia^66^ and also to be rich in cells expressing the neurotransmitters, dopamine and serotonin, that are associated with EtOH-related behaviors such as reward and locomotion^87–89^. These ectopic Hcrt neurons are also found to innervate the DP, a brain area that is not normally innervated by Hcrt neurons in larval zebrafish but is shown to participate in drug responses^90,91^ and thus may contribute to the EtOH-induced disturbances in behavior. These projections from the ectopic neurons to the SP and DP differ markedly from the projections from pAH Hcrt neurons that terminate in the LC and the RN, two areas rich in norepinephrine and serotonin, respectively, that are known to mediate sleep–wake and locomotor behaviors^92,93^. These alterations in Hcrt projections within the LC and RN may act as a potential mechanism underlying sleep disorders that are often comorbid with substance use disorders^94^, consistent with evidence showing Hcrt receptor antagonists to have clinical efficacy in improving sleep and reducing withdrawal symptoms^95^. A functional relationship between Hcrt and the LC is demonstrated in a study where optogenetic activation of Hcrt neurons in zebrafish stimulates locomotor activity and activates LC neurons expressing dopamine beta hydroxylase, while knockout of Hcrt neurons blocks this behavioral change^96^. A similar relationship between Hcrt and the RN is shown in rodents, with injection of Hcrt into the RN producing an increase in locomotor activity^97^. This provides support for a positive relationship between the increased innervation of the LC and RN by pAH Hcrt neurons shown here in zebrafish and increase in locomotor activity shown to be induced by embryonic EtOH exposure in zebrafish^9^. Although the effects of EtOH on Hcrt and Dyn reported here are all observed at 6 dpf before zebrafish sex can be determined^98^ and require further studies to determine if they are age dependent or sexually dimorphic, there is evidence showing that the effects of embryonic EtOH exposure on behavior and the EtOH-induced increase in Hcrt neurons observed in both zebrafish and rats persist into adulthood and are also sexually dimorphic, with females more affected than males^2,30,69^.

In summary, this study demonstrates that the distinct subpopulations of Hcrt neurons within the pAH, aAH and POA differ markedly under control conditions as well as in response to EtOH. The results show that embryonic EtOH exposure increases the number of Hcrt neurons that might lack inhibitory control by Dyn in the aAH and ectopically expressed Hcrt neurons in the POA, areas shown to have higher proliferative activity and higher Cxcl12 levels. In addition, EtOH stimulates the branching and terminal points of long projections to distinct brain areas with very different behavioral functions, including the LC and RN from pAH neurons and the SP from aAH neurons ectopically expressed in the POA. These findings support the idea that these distinct Hcrt subpopulations have markedly different behavioral functions and thus contribute very differently to the behavioral disturbances produced by embryonic EtOH exposure.

## Supplementary Information

Supplementary Tables.

## Data Availability

The datasets used and/or analyzed during the current study are available from the corresponding author on reasonable request.
