# Supplementary material for: Subpopulations of hypocretin/orexin neurons differ in measures of their cell proliferation, dynorphin co-expression, projections, and response to embryonic ethanol exposure
Source: Sci Rep. 2023 May 25;13:8448. doi: 10.1038/s41598-023-35432-w (PMC10213024; doi:10.1038/s41598-023-35432-w)
Supplement: Supplementary file 1 — Supplementary Tables. [file 41598_2023_35432_MOESM1_ESM.docx]

SUPPLEMENTARY INFORMATION

**Subpopulations of hypocretin/orexin neurons differ in measures of their cell proliferation, dynorphin**

**co-expression, projections, and response to embryonic ethanol exposure**

Nushrat Yasmin^1^, Adam D. Collier^1^, Olga Karatayev^1^, Abdul R. Abdulai^1^, Boyi Yu^1^, Milisia Fam^1^, Nailya Khalizova^1^,

Sarah F. Leibowitz^1*^

^1^Laboratory of Behavioral Neurobiology, The Rockefeller University, New York, NY

*Address for Correspondence:

Sarah F. Leibowitz,

Laboratory of Behavioral Neurobiology,

The Rockefeller University,

1230 York Avenue, New York, NY 10065, USA.

Phone: 212-327-8378, Fax: 212-327-8447,

E-mail: [leibow@rockefeller.edu](mailto:leibow@rockefeller.edu)

**Supplementary Table S1.** Effects of 0.5% EtOH on the number of Hcrt neurons and the density of pDyn transcripts in the pAH, aAH and POA of 6 dpf zebrafish, as shown by Two-Way ANOVA main effects and interactions and results from Post-Hoc tests.

| **Two-Way ANOVA** | | | | | | | | | | | | |
| --- | --- | --- | --- | --- | --- | --- | --- | --- | --- | --- | --- | --- |
| **Main Effects** | | **Embryonic EtOH** | | | | |  | **Brain Areas** | | | | |
|  |  | ***df*** | **F** | | ***p* Value** | |  | ***df*** | **F** | | ***p* Value** |  |
| Number of Hcrt Neurons | | 1,16 | 10.89 | | **0.0045**** | |  | 1,16 | 20.77 | | **0.0003***** |  |
| Density of pDyn Transcripts | | 1,26 | 2.965 | | 0.0969 | |  | 2,26 | 12.69 | | **0.0001***** |  |
| **Two-Way Interactions** | | **Embryonic EtOH x Brain Areas** | | | | |  |  |  | |  |  |
|  |  | ***df*** | **F** | | ***p*** | |  |  |  | |  |  |
| Number of Hcrt Neurons | | 1,16 | 23.73 | | **0.0002***** | |  |  |  | |  |  |
| Density of pDyn Transcripts | | 2,26 | 0.03605 | | 0.9646 | |  |  |  | |  |  |
| **Post-Hoc Tests** | | | | | | | | | | | |  |
| **Brain Areas** | **Measures** | | | **Control** | | **EtOH** | | | | ***p* Value** | |  |
| pAH | Number of Hcrt Neurons | | | 8.400 ± 0.400 | | 7.400 ± 0.600 | | | | 0.6313 | |  |
|  | Density of pDyn Transcripts | | | 94.927 ± 16.183 | | 119.641 ± 8.871 | | | | 0.9964 | |  |
| aAH | Number of Hcrt Neurons | | | 8.200 ± 0.200 | | 13.400 ± 1.030 | | | | **0.0001***** | |  |
|  | Density of pDyn Transcripts | | | 146.859 ± 30.826 | | 173.372 ± 14.828 | | | | 0.9926 | |  |
| POA | Number of Hcrt Neurons | | | NA | | 2.167 ± 0.307 | | | | NA | |  |
|  | Density of pDyn Transcripts | | | 69.684 ± 7.967 | | 88.005 ± 13.294 | | | | 0.9996 | |  |
| *Significant effects are boldface. **p<0.01; ***p<0.001.*  *Post-hoc data are represented as mean ± SEM.*  *Abbreviations: Hcrt:Hypocretin, EtOH: ethanol, pDyn: prodynorphin, pAH: posterior part of the anterior hypothalamus, aAH: anterior part of the anterior hypothalamus, POA: preoptic area.* | | | | | | | | | | | |  |

**Supplementary Table S2.** Effects of 0.5% EtOH on the density of Hcrt projection branch and terminal points to pAH and aAH projection areas in 6 dpf zebrafish, as shown by mean and SEM data and T-Test results.

| **Mean and SEM data** | | | | | | | | |
| --- | --- | --- | --- | --- | --- | --- | --- | --- |
| **Projection Areas of Hcrt** | |  | **Density of Projection Branch Points** | | | **Density of Projection Terminal Points** | | |
|  |  |  | **Control** | **EtOH** |  | | **Control** | **EtOH** |
| pAH Hcrt Neurons | pAH |  | 4.664 ± 0.982 | 4.467 ± 1.005 |  | | 8.408 ± 1.628 | 8.802 ± 1.647 |
|  | IH |  | 5.888 ± 2.010 | 3.064 ± 0.396 |  | | 8.472 ± 2.241 | 5.002 ± 0.638 |
|  | PT |  | 2.305 ± 1.362 | 9.662 ± 1.672 |  | | 5.052 ± 2.124 | 16.309 ± 1.520 |
|  | LFB |  | 6.649 ± 1.638 | 11.709 ± 1.447 |  | | 9.789 ± 2.039 | 16.018 ± 1.520 |
| aAH Hcrt Neurons | aAH |  | 7.685 ± 1.388 | 3.180 ± 0.515 |  | | 12.677 ± 1.345 | 8.473 ± 1.767 |
|  | PT |  | 0.798 ± 0.494 | 1.595 ± 0.636 |  | | 1.418 ± 0.923 | 3.723 ± 0.904 |
|  | LFB |  | 7.385 ± 1.243 | 7.681 ± 1.237 |  | | 10.366 ± 1.365 | 11.194 ± 2.296 |

|  | |  | | | **T-Tests** | | | | | | | |
| --- | --- | --- | --- | --- | --- | --- | --- | --- | --- | --- | --- | --- |
| **Projection Areas of Hcrt** | | |  | **Density of Projection Branch Points** | | | | | | **Density of Projection Terminal Points** | | |
|  |  |  |  | ***df*** | | **T ratio** | ***p* Value** |  | ***df*** | | **T ratio** | ***p* Value** |
| pAH Hcrt Neurons | pAH | |  | 8 | | 0.1403 | 0.8919 |  | 8 | | 0.1702 | 0.8691 |
|  | IH | |  | 8 | | 1.379 | 0.2053 |  | 8 | | 1.489 | 0.1747 |
|  | PT | |  | 8 | | 3.411 | **0.0092**** |  | 8 | | 3.315 | **0.0106*** |
|  |  |  |  |  |  |  |  |  |  |  |  |  |
|  | LFB | |  | 8 | | 2.315 | **0.0492*** |  | 8 | | 2.449 | **0.0399*** |
|  |  |  |  |  |  |  |  |  |  |  |  |  |
| aAH Hcrt Neurons | aAH | |  | 8 | | 2.618 | 0.0894 |  | 8 | | 1.873 | 0.0979 |
|  | PT | |  | 8 | | 0.9909 | 0.3507 |  | 8 | | 1.784 | 0.1123 |
|  | LFB | |  | 8 | | 0.1692 | 0.8698 |  | 8 | | 0.3098 | 0.7646 |

*Significant effects are boldface. *p<0.05; **p<0.01.*

*Mean data are represented as ± SEM.*

*Abbreviations: Hcrt:Hypocretin, EtOH: ethanol, pDyn: prodynorphin, pAH: posterior part of the anterior hypothalamus, aAH: anterior part of the anterior hypothalamus, IH: intermediate hypothalamus, PT: posterior tuberculum, LFB: lateral forebrain bundle.*
